# Changing paradigms in management of metastatic Castration Resistant Prostate Cancer (mCRPC)

**DOI:** 10.1186/1471-2490-14-55

**Published:** 2014-07-25

**Authors:** Eva Gupta, Troy Guthrie, Winston Tan

**Affiliations:** 1 Mayo Clinic, 4500 San Pablo Rd S, Jacksonville 32224, FL, USA; 2 Baptist Cancer Institute, Jacksonville, FL, USA

**Keywords:** Castration resistant prostate cancer, CYP17 inhibition, Androgen deprivation therapy, Abiraterone, Enzalutamide, Ketoconazole, Orteronel, ARN-509, Galeterone (TOK-001)

## Abstract

Recently, the standard of care for metastatic Castration Resistant Prostate Cancer (mCRPC) has changed considerably. Persistent androgen receptor (AR) signaling has been identified as a target for novel therapies and reengages the fact that AR continues to be the primary target responsible for metastatic prostate cancer. Androgen receptor gene amplification and over expression have been found to result in a higher concentration of androgen receptors on tumor cells, making them extremely sensitive to low levels of circulating androgens. Additionally, prostate cancer cells are able to maintain dihydrotestosterone (DHT) concentration in excess of serum concentrations to support tumor growth. For many years ketoconazole was the only CYP17 inhibitor that was used to treat mCRPC. However, significant toxicities limit its use. Newly approved chemotherapeutic agents such as Abiraterone (an oral selective inhibitor of CYP17A), which blocks androgen biosynthesis both within and outside the prostate cancer cells), and enzalutamide (blocks AR signaling) have improved overall survival. There are also ongoing phase III trials for Orteronel (TAK- 700), ARN- 509 and Galeterone (TOK-001), which targets androgen signaling. In this review, we will present the rationale for the newly approved hormonal treatments, their indications and complications, and we will discuss ongoing trials that are being done to improve the efficacy of the approved agents. Finally, we will talk about the potential upcoming hormonal treatments for mCRPC.

## Introduction

Prostate cancer is the most common cancer affecting men and represents the second leading cause of cancer related mortality in the western world [1]. In 1941, Huggins and Hodges et al. [2], demonstrated that androgen withdrawal led to regression of prostate cancer and alleviation of pain in these patients. This demonstrated the androgen dependence of normal prostate and prostate cancer cells for growth and survival.

The initial standard of care in many high-risk patients includes androgen deprivation therapy (ADT) [3,4] and radiation therapy. ADT can be achieved by either medical or surgical castration (bilateral orchidectomy) [5]. Castration reduces the serum testosterone to very low levels, which is known as the castration level. Until recently, medical castration was achieved by Gonadotropin-releasing hormone (GnRH) agonists. GnRH agonists inhibit the pituitary release of luteinizing hormone, which is necessary for testicular androgen production. Degarelix is a GnRH antagonist, which lowers androgen levels but causes an unacceptably high rate (40%) of local injection site reactions and has not found much favor in clinical practice. Anti-androgens, such as flutamide and bicalutamide, can block the interaction of testosterone and DHT with its receptor. Combination GnRH agonists and androgen blockers has been called total androgen blockade (TAB) and was popular in the 1990’s to treat metastatic prostate cancer. Despite total androgen blockade, prostate cancer is known to progress in 18 to 48 months and is referred to as castration resistant prostate cancer (CRPC). CRPC is characterized by elevated levels of prostate specific antigen PSA despite low levels of testosterone. Prostate cancer deaths are typically the result of metastatic castrate resistant prostate cancer (mCRPC), and historically, the median survival for men with mCRPC has been less than 2 years [6]. Randomized studies with TAB have failed to demonstrate improvement in overall survival (OS) [7]. This is thought to occur due to multiple escape mechanisms that fuel tumor growth [8]. Previously this was thought to be a hormone refractory state, but recently it has been recognized that androgen receptor expression is never lost. In the castration resistant state, androgen receptor gene amplification [9,10], alterations in expression of coactivators, and androgen receptor gene over expression have been found to result in higher concentrations of androgen receptors on tumor cells, making them extremely sensitive to low levels of circulating androgens. Prostate cancer cells have also been found to be able to maintain dihydrotestosterone (DHT) concentrations in excess of serum concentrations to support growth and proliferation [11]. They may also synthesize DHT de-novo [12] or convert adrenal steroids to DHT, which has five fold greater affinity than testosterone for the androgen receptor. In addition, selective mutations in the androgen receptor when exposed to anti-androgens may be responsible for resistance. Metastatic CRPC is an invariably fatal disease. Chemotherapy including docetaxel [13] as first-line, cabazitaxel as second-line, and active cellular immunotherapy with sipuleucel-T [14] has also not been found to produce a major survival improvement in mCRPC.

**Figure 1:**
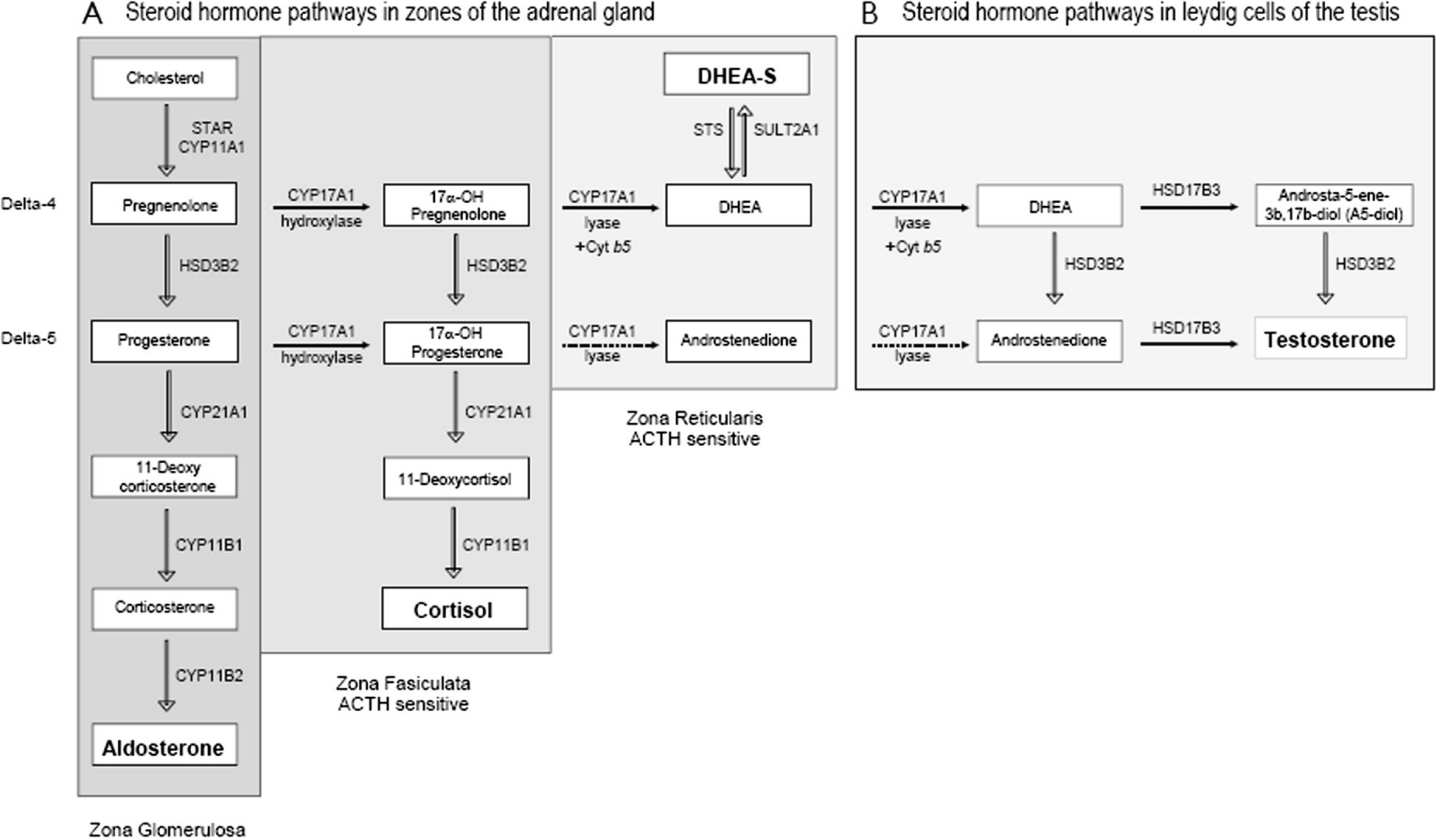
**Pathways of steroid synthesis. A**. Pathways of steroid synthesis in the adrenal gland. **B**. Pathways of steroid synthesis in leydig cells of testis.

Focus has now shifted to the inhibitors of steroid biosynthesis [15]. CYP17 is a cytochrome P450 enzyme [16] that catalyzes two key reactions involved in the production of sex steroids (Figure 1). The 17α-hydroxylase activity converts pregnenolone to 17α-hydroxypregnenolone, which is a major precursor of metabolism into mineralocorticoids, glucocorticoids and androgens Treatment with ketoconazole, which inhibits 17α-hydroxylase, leads to suppression of glucocorticoid and mineralocorticoid production and causes a secondary increase in pituitary ACTH. In addition to suppression of androgens, it has been shown to slow tumor activity. Ketoconazole is a non-steroidal imidazole anti-fungal agent with CYP17 inhibition that has been used off-label as second-line hormonal therapy for prostate cancer since the 1980s [17-20]. It is an inhibitor of testicular and adrenal androgen synthesis, and high doses have typically been used to suppress tumor activity. High dose ketoconazole (HDK) has been has shown to have PSA response, but no survival benefit has been shown [21]. It is also associated with potential and significant adverse events, including fatal hepatic dysfunction, adrenal insufficiency (bone fragility, hypotension, and hyperkalemia), nausea and vomiting, gynecomastia, QT prolongation, and potentially fatal drug interactions. In a trial to evaluate the efficacy of ketoconazole along with simultaneous anti-androgen withdrawal (AAWD) in 20 patients with CRPC, Small et al. found 55% had a greater than 50% fall in prostatic specific antigen (PSA) [22]. In another study of 50 patient [23], Small et al. demonstrated that patients who have progressive disease despite anti-androgen withdrawal also benefit from subsequent ketoconazole therapy. In a larger phase III study of HDK therapy [24] the authors randomized 260 patients to AAWD alone (n = 132), or together with oral Ketoconazole (400 mg tid) and hydrocortisone (30 mg by mouth each morning, 10 mg by mouth. each evening; n = 128). PSA response (27% vs. 11%) and objective response (20% vs. 2%) were significantly more in the ketoconazole group compared to AAWD alone, although there was no difference in survival. Androgen levels have been shown to decline with Ketoconazole therapy, but the levels then climb at the time of progression. Progressive disease while on Ketoconazole has been postulated due to an escape from HDK induced androgen suppression, and it highlights the need for more effective agents.

In addition to blocking CYP17 activity, ketoconazole also inhibits other important metabolizing enzymes, such as CYP3A and CYP24A1, suggesting that concomitant ketoconazole administration may alter drug exposure or pharmacokinetic variability. This necessitates careful monitoring of adverse events and drug interactions. The responses observed after treatment with ketoconazole lead to the investigation of stronger and more selective CYP 17 inhibitors with a more favorable toxicity profile than ketoconazole [25].

The last several years, has seen new drug development on the rational of targeted approaches based on a better understanding of the disease process. These have created a changing paradigm in the hormonal treatment of advanced prostate cancer.

## Review

### New approved hormonal treatments

Recently two new hormonal therapy agents have been approved by the US Food and Drug Administration (FDA) for the treatment of patients with mCRPC: Abiraterone acetate (Zytiga) [26] and enzalutamide previously known as MDV3100 (now called Xtandi) [27].

**Abiraterone (Zytiga)** is an oral, selective and potent irreversible inhibitor of CYP17A, which is an enzyme that catalyzes both 17 alpha-hydroxylase and 17, 20-lyase reactions. It blocks androgen biosynthesis both within and outside of the prostate gland. It was first found to be efficacious in Phase I-II studies [28] of castrate-resistant prostate cancer. It was also tested in the treatment of patients with CRPC, who are either chemotherapy naive or have received prior therapy with docetaxel [29,30]. Abiraterone decreases the production of androgens by the adrenals, prostate, and also within the tumor cells. Evidence from phase I and phase II studies [31,32] demonstrated that Abiraterone suppresses the serum androgen levels and achieves PSA and clinical responses in chemotherapy naïve and docetaxel pretreated patients with mCRPC. Phase II and III studies have used a 1000 mg/day dose, although the maximum tolerated dose was 2000mg/day. Abiraterone was generally well tolerated. Hypokalemia (88%), hypertension (40%) and fluid overload (13%) were the most common adverse events noted.

A large randomized controlled phase III trial of 1195 patients (COU-AA-301) [33] comparing Abiraterone-prednisone vs. placebo-prednisone had to be terminated early (median survival 12.8 months) when the study met planned primary outcomes at the time of interim analysis. Patients with prior ketoconazole treatment for prostate cancer and a history of adrenal gland or pituitary disorders were excluded in this trial. The OS rate favored abiraterone (14.8 months vs. 10.9 months). Secondary end points, including time to PSA progression (10.2 vs. 6.6 months; P < 0.001), progression-free survival (5.6 months vs. 3.6 months; P < 0.001), pain palliation (44% vs. 2%), and PSA response rate (29% vs. 6%, P < 0.001) favored the treatment group. Mineralocorticoid-related adverse events, including fluid retention (31% vs. 22% placebo; P < 0.001) and hypokalemia (17% vs. 8% placebo), were more frequently reported in the Abiraterone acetate–prednisone group than in the placebo–prednisone group. There was a non-significant increase in grade 1–2 cardiac events in the treatment group (13% vs. 11% placebo). Seventy percent of patients in this trial had received one prior chemotherapy regimen, and 30% had been treated with two prior chemotherapeutic regimens. Abiraterone acetate is now considered standard of care for patients following chemotherapy. This study led to the approval of Abiraterone acetate for docetaxel pretreated CRPC in April 2011.

In December 2012, Abiraterone in combination with prednisone received FDA approval for treatment of mCRPC in chemotherapy naïve patients as well. In a phase III randomized controlled trial (COU-AA-302) [34], 1088 patients with mCRPC who had not received chemotherapy were assigned either to abiraterone and prednisone (N = 546) or placebo plus prednisone (N = 542). The primary endpoints were radiographic progression free survival (rPFS) and overall survival (OS). The study was unblinded after a planned interim analysis, which was performed after 43% of the expected deaths had occurred. Abiraterone improved rPFS (16.5 months vs. 8.3 months, HR 0.53; 95% CI 0.45-0.62; P < 0.001). It also showed a trend towards improved OS (median not reached, vs. 27.2 months for prednisone alone; HR 0.75; 95% CI, 0.61 to 0.93; P = 0.01). Abiraterone–prednisone showed superiority over prednisone alone with respect to time to initiation of cytotoxic chemotherapy (25.2 vs. 16.8 months, p-value <0.001) opiate use for cancer-related pain (not reached vs. 23.7 months, p-value <0.001), prostate-specific antigen progression (11.1 vs. 5.6 months, p-value <0.001), and decline in performance status (12.3 vs. 10.9 months, p-value 0.005).

**Toxicity profile**- the main adverse events of Abiraterone are related to excess mineralocorticoid, which includes fluid retention (33%) and hypokalemia (18%). This is due to the inhibition of 17 alpha hydroxylase, which causes a compensatory rise in ACTH. Abiraterone should be administered with prednisone daily and monthly potassium and blood pressure monitoring is essential during treatment. While co-administration of prednisone is manageable, long term use in earlier disease phases could be problematic due to the potential adverse events. These include diabetes, weight gain, Cushing syndrome and osteoporosis. Fatigue, joint swelling, edema, cough, vomiting, elevated liver enzymes, hyperglycemia and hypercholesterolemia have also been reported.

In a recent retrospective study, Peer et al. [35] found abiraterone to be superior to ketoconazole in the treatment of docetaxel refractory mCRPC. PSA response was 46% in the abiraterone group vs. 19% in the ketoconazole group (OR 4.3, P = 0.04), median biochemical progression free survival (PFS) 7 vs. 2 months (HR 1.54, P = 0.02), median radiological PFS 8 vs. 2.5 months (HR 1.8, P = 0.043), median OS 19 vs. 11 months (HR 0.53, P = 0.79) and treatment interruption due to severe adverse events 8% (n = 2) versus 31% (n = 8) (0R 0.6, P = 0.023).

### Enzalutamide (Xtandi)

Enzalutamide (formerly MDV300) is an oral, second-generation androgen receptor antagonist that competitively inhibits androgen binding to the AR. In contrast to the first generation anti-androgens such as flutamide and bicalutamide, enzalutamide binds to the receptor with greater affinity [36]. In the setting of increased AR expression, bicalutamide is associated with AR recruitment to enhancer regions and aberrant recruitment of coactivators to these transcription complexes, leading to target gene activation rather than repression [37]. Enzalutamide does not display agonism in AR-overexpressing cells and this may explain its increased molecular efficacy. Enzalutamide may induce a conformational change in AR distinct from that induced by bicalutamide making it more efficacious in inhibiting the translocation of AR to the nucleus and its DNA interaction. In a phase I-II study [36], 140 men including 78% with mCRPC received doses ranging from 30-600mg daily. Half of the patients had previously received chemotherapy and three-fourths had received at least two lines of hormonal therapy. PSA responses were observed in 62% of the chemotherapy naïve patients and 51% in docetaxel treated patients [36]. 22% of the patients had a soft tissue response and 56% of the patients with bone disease had stabilized bone disease. The maximum tolerated dose was determined to be 240 mg daily. The median rPFS was 56 weeks and 24 weeks in the chemotherapy naïve and the chemotherapy pretreated group, respectively.

Enzalutamide was approved after the publication of a phase III [37], double-blind placebo-controlled randomized trial by Scher et el (AFFIRM TRIAL) in which 1199 men with mCRPC were randomized after chemotherapy to placebo vs. oral enzalutamide at a dose of 160 mg per day. The median OS was 18.4 months in the enzalutamide group versus 13.6 months in the placebo group (P < 0.001). The secondary endpoints including the PSA- level response rate (54% in the enzalutamide group vs. 2% in the placebo group), soft tissue response rate (29% vs. 4%), the time to PSA progression (8.3 months in the enzalutamide group vs. 3 months in the placebo group), rPFS (8.3 months in the enzalutamide group vs. 2.9 months in the placebo group), time to the first skeletal event (16.7 months in the enzalutamide group vs. 13.3 months in the placebo group) quality of life response rate (43% in the enzalutamide group vs. 18% in the placebo group) pain palliation achieved in (45% in the enzalutamide group vs. 7% in the placebo group) showed significant improvement in the enzalutamide group. The enzalutamide group had higher incidence of fatigue, hot flashes, musculoskeletal pain and headaches. Rates of hyperglycemia, weight gain and glucose intolerance were not different between the two groups. Cardiac disorders were seen in 6% of the patients receiving enzalutamide and 8% in the placebo group. Hypertension was seen in 6.6% in the enzalutamide group vs. 3.3% in the placebo group. Seizures were reported in 0.6% in the enzalutamide group vs. placebo.

The results of the large phase III randomized trial (PREVAIL trial) [38] were recently presented at the 2014 Genitourinary Cancer Symposium. This trial evaluated enzalutamide against placebo in chemotherapy naïve men with mCRPC. In the study, 1,717 chemotherapy naïve patients with mCRPC were assigned to receive 160 mg/day of enzalutamide vs. placebo in a double blind fashion. After a median follow-up of 20 months, interim analysis showed that enzalutamide significantly reduced the risk of death by 29% (HR 0.706, 95% CI 0.60-0.84, p <0.0001) and decreased the risk of radiographic progression by 81% (HR 0.186,95% CI 0.15-0.23, P < 0.0001). 59% of the patients in the enzalutamide group had a soft tissue response compared with 5% in the placebo arm. Enzalutamide also delayed the median time to chemotherapy initiation by 17 months as compared to placebo. The patients on the placebo arm needed to start cytotoxic chemotherapy after a median of 10.8 months due to disease progression. Median time to PSA progression was 2.8 months in the placebo group vs. 11.2 months in the enzalutamide group. The adverse effects included grade 1–2 fatigue (36% vs. 26%), back pain (27% vs. 22%) constipation (22% vs. 17%) and arthralgia (20% vs. 16%) in the enzalutamide vs. placebo group. The patients with a history of seizure disorders were excluded from the trial.

The results of the PREVAIL data will be submitted to the FDA for approval. If enzalutamide gets approval, there will be more choices available to treat chemotherapy naïve patients with mCRPC.

**Toxicity profile**- enzalutamide is reported to cause fatigue (11%), hot flashes (20%), headache (12%), nausea, diarrhea, constipation and musculoskeletal pain. Other reported adverse events include hyperglycemia, weight gain and glucose intolerance. Seizure was reported in 0.6% of the enzalutamide group at 360 to 600 mg doses. Thus the maximum tolerated dose (MTD) is 240 mg/day (Table 1).

**Table 1:** Newly approved hormonal agents for the treatment of mCRPC

**Drug**	**Date of FDA approval and indication**	**Mechanism of action**	**Side effects**
Abiraterone acetate (Zytiga)	December2012- (COU-AA-301)	An androgen biosynthesis inhibitor of 17 alpha hydroxylase/C-17,20-lyase within prostate cancer cells and outside	Fatigue, joint swelling, edema, hot flashes, diarrhea, cough.
	In combination with prednisone for treatment of patients in mCRPC [29]		
			Administration of prednisone is necessary to overcome hypertension, hypokalemia, fluid overload from mineralocorticoid excess induced by CYP17-inhibition
	April 2012- (COU-AA-302) [30]		
	Treatment of mCRPC in patients with have received prior chemotherapy containing Docetaxel		
Enzalutamide (Xtandi) Previously known as MDV3100	August 2012- AFFIRM trial [34]	Androgen receptor inhibitor- inhibits multiple steps in AR signaling	Fatigue, hot flashes, musculoskeletal pain, hyperglycemia, weight gain, Seizures in 0.6% of the patients.
	Monotherapy for mCRPC who have previously received Docetaxel		
	January 2014- PREVAIL trial [35]		
	Survival benefit in chemotherapy naïve patients. Awaiting FDA approval.		

### New drugs under development

**Orteronel (TAK 700)** - is a non-steroidal, selective inhibitor of 17, 20 lyase, which is involved in androgenic steroid production. Selective inhibition improves its toxicity profile as compared to CYP17 inhibition. Orteronel causes less treatment related adverse events. In phase I and II studies [39], Orteronel given in twice daily doses of 100, 200,300,400 and 600 mg was well tolerated. The most common adverse events were gastrointestinal toxicity and grade 3 fatigue. At 12 weeks, the median DHEA-S and testosterone levels decreased from baseline in all the groups [39]. The mean number of circulating tumor cells decreased from 16.6 (per 7.5 ml blood) at baseline to 3.9 at 12 weeks.

The results of a large randomized, double blind, multicenter phase III study (ELM-PC4) was presented at the ASCO symposium in January 2014. The results showed that there was no improvement in OS with orteronel + prednisone vs. placebo in patients with mCRPC that progressed during or following chemotherapy. However there was improvement in rPFS over the control arm.

Currently the Radiation Therapy Oncology Group (RTOG) has a trial using TAK/orteronel in addition to conventional LHRH agonist to test if it will improve overall survival. The southwest oncology group (SWOG) is conducting a trial to compare overall survival in newly diagnosed metastatic prostate cancer patients who were randomly assigned to androgen deprivation therapy (ADT) + TAK-700 vs. ADT + bicalutamide.

**ARN509**- is a small molecule that is structurally similar to enzalutamide. It inhibits both AR nuclear translocation and AR binding to DNA [40]. In contrast to bicalutamide, it exhibits no agonist activity in prostate cancer cells that over express AR. In a phase I study [41] of men with mCRPC, it was shown to have an excellent safety profile at 240 mg/day. Preliminary results were reported by the Prostate Cancer Working Group in 2013 at the GU cancer symposium [42]. Among 46 men with mCRPC, 26 were treatment naïve and 21 had prior treatment with abiraterone. At 12 weeks, the PSA response was 88% in the treatment naïve and 29% in the prior-treatment group. The toxicity profile included fatigue (38%), nausea (29%) and pain (24%). Currently, a phase II multicenter study (NCT01171898) is evaluating the activity of ARN-509 in three different populations of men with mCRPC (high risk non-metastatic CRPC, metastatic treatment naïve CRPC and progressive disease after abiraterone acetate) and further phase III trials are planned.

**Galeterone (TOK-001)** - is another addition to the next generation androgen receptor antagonists and CYP17A1 inhibitors. It works by disrupting multiple androgen signaling pathways simultaneously and by down regulating the androgen receptor [43,44]. ARMOR 1 [45] was a multicenter dose escalation study of Galeterone for the treatment of chemotherapy naïve non-metastatic prostate cancer and mCRPC. The data from ARMOR 1 were presented at the 2012 AACR and 2012 ASCO meetings, showed that the drug is well tolerated. ARMOR2 is an ongoing phase II multicenter trial to evaluate the efficacy and safety of Galeterone in the following populations - metastatic treatment naïve patients, non-metastatic treatment naïve patients, patients who have progressed on Abiraterone and patients who have progressed on Enzalutamide. The primary endpoints of the study are reduction in PSA levels and safety. The secondary endpoints include tumor response by the Response Evaluation Criteria in Solid Tumors (RECIST), AR modulation and levels of circulating tumor cells and markers of CYP17lyase inhibition (Table 2).

**Table 2:** Newer agents under development for the treatment of mCRPC

**Agent**	**Mechanism of action**	**Phase of development**	**Side effects**	**Ongoing trials**
Orteronel (TAK-700)	Non-steroidal, selective inhibitor of 17, 20lyase, an enzyme required for androgen biosynthesis.	The results of Phase III trial (ELM-PC 4) did not show any survival benefit in chemotherapy naïve patients. An improvement in rPFS was seen.	Fatigue, GI toxicity	RTOG and SWOG- TAK + LHRH agonist to test whether improvement in OS or not
Galeterone (TOK-001)	Next generation AR antagonist and CYP17A1 inhibitor	ARMOR 1- phase I study showed the drug is well tolerated [42]	Fatigue, Nausea, Diarrhea	ARMOR 2 is underway in 4 distinct populations
				1. Metastatic and treatment naïve
				2. Non metastatic and treatment naïve
				3. Patients who have progressed on abiraterone
				4. Patients who have progressed on enzalutamide
ARN-509	Inhibits AR translocation and AR binding to DNA, does not exhibit agonist properties in the context of AR over-expression	Results from phase I studies showed that the drug is well tolerated and PSA decline at 12 weeks (>50% from the baseline) were observed in 46.7% of the patients. [38]	Fatigue, nausea, pain	Phase II study is underway in patients with mCRPC (NCT01171898)

### Treatment sequencing and combination therapy

The last decade has seen tremendous progress in prostate cancer research and has led to a better understanding of prostate cancer biology. This understanding has led to multiple new drugs that have been approved and have shown a survival benefit in patients with metastatic disease. With the approval of abiraterone and enzalutamide for castrate resistant prostate cancer in the post chemotherapy setting and abiraterone in the chemotherapy naïve state, there is an emerging theme of questions on how we use the new drugs sequentially. We would like to propose a schema that might help the clinician, but the ultimate answer would only be provided by randomized clinical trials. What we know based on the trials include the following:

### Chemotherapy naïve

Sipuleucel-T- (Provenge) - immunotherapy

Abiraterone

Docetaxel

### Post chemotherapy- docetaxel

Cabazitaxel- chemotherapy

Abiraterone

Enzalutamide

### Symptomatic bone metastasis

Radium 223 (Xofigo)

What we do not know is to whom we should give chemotherapy first or if we should give chemotherapy after initial therapies have failed. Clinically, physicians are giving sipuleucel-T or abiraterone first followed by chemotherapy. For those with significant visceral disease and aggressive presentation most would start with docetaxel. If enzalutamide is approved in the chemotherapy naïve patients, which drug would become the first line of treatment, enzalutamide or abiraterone? We would need to do randomized trials of sequential treatments to answer these questions.

When the data of ECOG 3809 (randomized trial of chemotherapy- docetaxel for 6 cycles plus leuprolide and bicalutamide or hormone therapy alone) is published, should we start patients with metastatic disease with hormone therapy plus chemotherapy upfront? Although, it is good to have a plethora of treatment options today, there appears to be more questions than answers.

## Conclusion

It is amazing that we have turned around in the past few years from calling progressive metastatic prostate cancer-hormone refractory to castrate resistant disease. We now realize that the optimal hormone suppression in the past was not adequate. With a variety of better androgen receptor blockers and targets, we are now at a point where we can continue to improve the efficacy of these agents. Should we stop LHRH agents once we use these agents, what is the ideal testosterone level that we need to achieve, what level would correlate with response, are there markers that are better than testosterone and many more? Future research should be directed towards optimizing efficacy through less toxic combinations and should ultimately make a difference in improving the survival and quality of life of our patients.

## Competing interests

The authors declare that they have no competing interests.

## Authors’ contributions

EG, TG and WT contributed equally to this article. All authors read and approved the final manuscript.

## Pre-publication history

The pre-publication history for this paper can be accessed here:

http://www.biomedcentral.com/1471-2490/14/55/prepub
